# Case Report: Sclerosing angiomatoid nodular transformation of the spleen: surgery or conservative treatment?

**DOI:** 10.3389/fimmu.2025.1530363

**Published:** 2025-07-28

**Authors:** Shuai Yan, Zihan Wang, Jiajie Lu, Liuxia Yuan, Linling Ju, Huixuan Wang, Lin Chen, Weihua Cai, Feng Xiao, Jinzhu Wu

**Affiliations:** ^1^ Medical School of Nantong University, Nantong University, Nantong, Jiangsu, China; ^2^ Institute of Liver Diseases, Affiliated Nantong Hospital 3 of Nantong University, Nantong, Jiangsu, China; ^3^ Department of Hepatobiliary Surgery, Affiliated Nantong Hospital 3 of Nantong University, Nantong, Jiangsu, China; ^4^ Department of Pathology, Affiliated Nantong Hospital 3 of Nantong University, Nantong, Jiangsu, China

**Keywords:** sclerosing angiomatoid nodular transformation of the spleen, sant, spleen, case report, cancer

## Abstract

Sclerosing angiomatoid nodular transformation (SANT)is a rare vascular sclerosing mass-like lesion, often discovered incidentally during routine imaging evaluation or during visits for the patient’s primary disease. SANT has complex pathological manifestations, unknown natural course of development and rarity, which makes it difficult for clinicians and pathologists to define its true nature. We report a 55-year-old male patient who came to see a doctor due to a physical examination accidentally found a spleen mass. He had hypertension and hyperlipidemia, and had no obvious clinical symptoms. After that, he underwent surgical resection of the spleen, and the patient had no special discomfort after treatment. Our results show that surgical resection is an efficient treatment for SANT patients without obvious clinical symptoms, but the patient ‘s comprehensive conditions should also be considered.

## Graphical Abstract

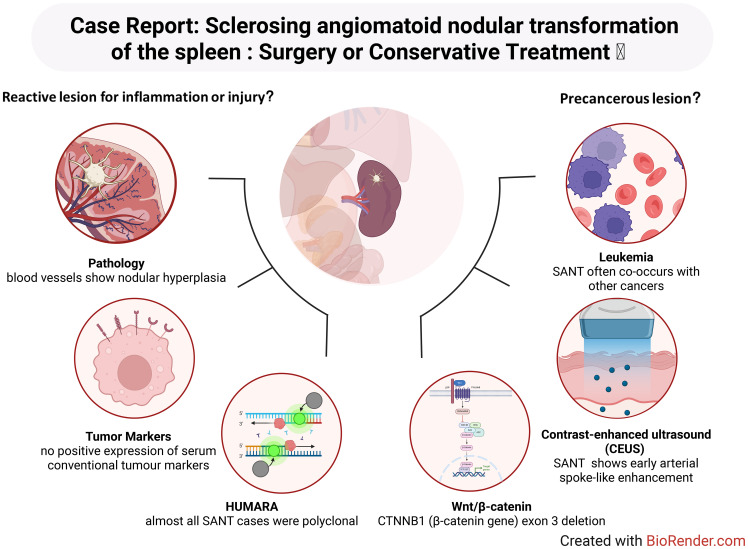

## 1 Introduction

Sclerosing angiomatoid nodular transformation (SANT) is a rare vascular sclerosing mass-like lesion that is often found by chance during a routine imaging evaluation or during the visit for the patient’s primary disease (1, 2). The existing debate surrounding SANT involves its classification as a benign reactive lesion related to inflammation or vascular injury (1, 3), a precancerous lesion potentially associated with malignant conditions or genetic mutations (4, 5), or as a possible IgG4-related entity or inflammatory pseudotumor (IPT)-like lesion (6); this lack of consensus often leads to ambiguity in clinical decision-making regarding optimal treatment approaches. Therefore, We report a patient with spleen SANT without obvious discomfort and review two opposing mainstream views on the mechanism of SANT. At present, no literature has discussed this point.

## 2 Case presentation

A 55-year-old male was referred for evaluation after a splenic mass was discovered during a routine physical examination. He is a company employee and had no history of exposure to industrial poisons, dust, or radioactive substances. He had a history of smoking and no special family history. He had high blood pressure and diabetes.

**Figure 1:**
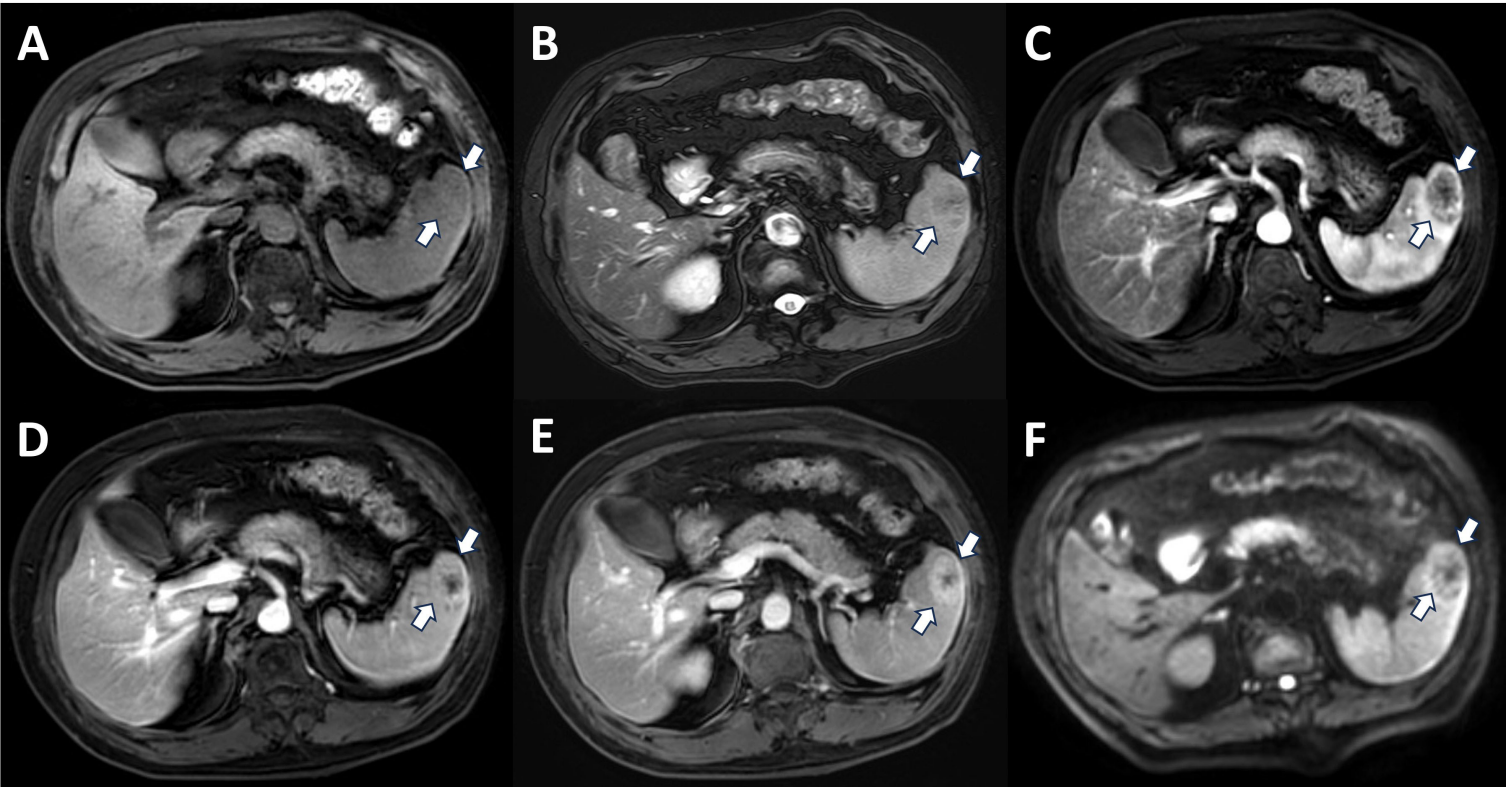
MRI of the patient’s spleen. T1WI showed a mass of fuzzy low-signal shadows on the upper pole of the spleen **(A)**. The lesion site on T2WI was clearer, showing a slightly oval low-signal shadow **(B)**. On enhanced MRI, the edge of the lesion was enhanced, and the punctate enhancement was obvious. Irregular patchy low-signal shadows were observed in the center of the lesion, and delayed patchy enhancement was observed inside the lesion in the portal phase and delayed phase **(C-E)**. On DWI, the lesion site showed a spoke wheel pattern sign of low signal in the center, and the edge showed irregular punctate enhancement **(F)**. MRI, magnetic resonance imaging; T1WI, T1 weighted image; T2WI, T2 weighted image; DWI, diffusion weighted imaging.

His blood pressure fluctuated between 180-114/105–60 mmHg. The blood glucose level fluctuated between 8.12-22.82 mmol/L. The laboratory examination showed high triglyceride (5.96 mmol/L, normal value 0.48-1.88 mmol/L), high vascular endothelial growth factor (VEGF) (211.05 pg/ml, normal value 0–160 pg/ml), and no other abnormalities. In the follow-up routine and enhanced MRI examination of the patient, it was found that the spleen had a round spoke wheel pattern with a clear boundary and showed low signal in T2WI and DWI and delayed enhancement in the enhanced scan (**Figure 1**).

Considering the uncertainty of the splenic lesion and to prevent the rupture of large blood vessels and the spread of malignant tumours, we did not perform endoscopic ultrasound-guided fine needle aspiration cytology (EUS-FNA). After that, the patient underwent laparoscopic splenectomy, the operation was successful, and no complications occurred. The patient was discharged 7 days later.

**Figure 2:**
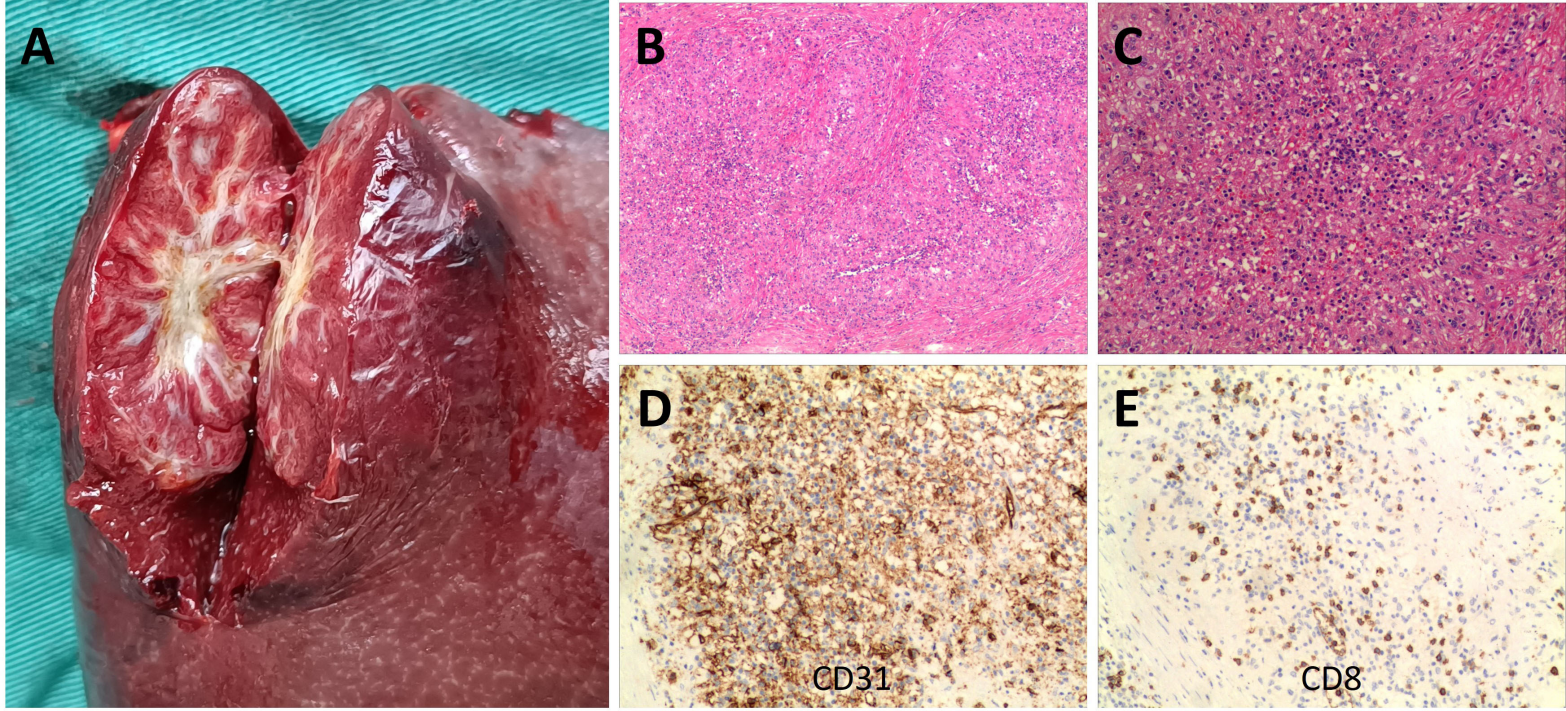
Patient surgical specimens and pathological results. A well-defined 4 cm mass is shown in the 13 x 9 x 6 cm spleen section, in which multiple red nodules delineated by dense white fibres and scar tissue are visible **(A)**. Under the microscope, this case showed capillary-type vascular spaces. The lesion was composed of scattered round and oval hemangioma-like nodules, with fibrosclerotic stroma between the nodules [**(B)** hematoxylin and eosin staining, original magnification ×40]. Honeycomb vascular lacunae could be seen in the nodules [**(C)** hematoxylin and eosin staining, original magnification ×100]. CD31 labelling revealed positive vascular spaces within the nodule [**(D)** original magnification ×100]. CD8 labelling revealed negative vascular spaces within the nodule [**(E)** original magnification ×100].

The pathological analysis of the surgical specimen showed that the section of the spleen with a size of 13 * 9 * 6 cm showed a well-defined 4 cm mass with multiple red nodules delineated by dense white fibres and scar tissue (**Figure 2A**). Microscopically, this case showed capillary-type vascular lacunae. The lesion was composed of scattered round and oval hemangioma-like nodules, with fibrous sclerosing stroma between the nodules (**Figure 2B**) and honeycomb-like vascular lacunae in the nodules (**Figure 2C**). CD31 labelling showed a positive vascular space in the nodules (**Figure 2D**). CD8 labelling showed a negative vascular space (**Figure 2E**). The other immunohistochemical results were CD34 (+), ERG (+), CD38 plasma cells (+), CD68 (+), Ki67 (~ 5% +), Vimentin (+), SMA (+), P53 (–), and IgG4 (+). The final pathological diagnosis was SANT.

No recurrence or metastasis was found during the 25-month follow-up.

## 3 Discussion

### 3.1 Concept and clinical features of SANT

SANT was first described by Martel et al. (4) in 2004 through a seminal report of 25 unique splenic lesions. In this pioneering study, SANT was characterized as a “benign splenic lesion with a distinctive morphology” (7). Since the initial description, only approximately 170 additional cases have been documented in the literature to date (8), reinforcing the notion that SANT is an exceedingly rare clinical entity with an incidence well below 1% of all splenic tumours. As demonstrated in the documented case series, SANT primarily affects middle-aged adults, with the majority of cases occurring between the ages of 30 and 60 years (8). While some reports have observed a slight female predominance, this gender bias has not been consistently confirmed across all studies (2, 9). Clinically, patients with SANT are usually asymptomatic, and the lesions are frequently discovered incidentally during imaging examinations performed for unrelated reasons, as was the case with the patient in question (10). In the event of symptoms manifesting, which occurs in a limited number of cases, these are typically non-specific and characterised by mild left upper abdominal pain or discomfort, primarily attributable to the mass effect of the splenic lesion (11). It is noteworthy that SANT lesions exhibit significant variability in size. The range of reported diameters is broad, extending from several millimetres to 17 centimetres. However, the majority of lesions are approximately 3–8 centimetres at the time of diagnosis (12). Importantly, there have been no definitive clinical observations suggesting metastatic potential or malignant transformation associated with SANT. The clinical course of SANT is consistently benign, with complete resolution following splenectomy and no recurrence documented during follow-up in the reported cases (4, 13).

### 3.2 Pathological perspectives of SANT

As previously mentioned, the clinical manifestations of SANT are non-specific. However, at the microscopic level, the pathological features of SANT are distinct, clearly differentiating it from other splenic lesions. Macroscopically, SANT presents as a solitary, well-circumscribed mass within the spleen, typically characterized by a multinodular cut surface (7, 14). Martel et al. (4) provided a vivid description of the gross pathology, characterising it as clusters of cohesive, reddish-brown nodules separated by dense fibrous stroma. Microscopically, these nodules comprise proliferative vascular channels, which display a distinctive admixture of slit-like capillaries, larger sinusoid-like vessels, and small vein-sized vessels, typically encased by concentric rings of collagen fibres (“onion-skin” fibrosis) (4, 15–17). Scattered within the fibrotic stroma are inflammatory cells (lymphocytes and plasma cells) and hemosiderin-laden macrophages, reflecting previous haemorrhage and reparative processes (4, 18–21). Notably, at the cellular level, SANT lesions exhibit very regular nuclear morphology under microscopic observation, characterized by uniform nuclear size, minimal to no nuclear atypia (which implies no abnormal variations in nuclear size, shape, or chromatin distribution), and rare mitotic figures (4, 22–24). These cellular characteristics provide compelling evidence to support the pathological diagnosis of SANT as a benign entity, suggesting limited proliferative activity or absence of malignant growth potential. Due to its unique yet complex histological features, several hypotheses and interpretations regarding the nature of SANT have been proposed in earlier studies. It has been hypothesised by certain scholars that SANT could be an atypical variant of splenic hamartoma. This condition is characterised by the presence of benign lesions composed of normal splenic constituents, arranged in an aberrant architecture. Alternatively, SANT was regarded as a distinct subtype of inflammatory pseudotumour, a benign lesion arising from chronic inflammatory or reparative processes. However, the distinctive histological presentation of SANT, particularly the prominent “onion-skin” collagenous fibrosis surrounding vascular nodules comprised of three distinct vessel types (capillary-like, venular, and sinusoidal), clearly distinguishes it from conventional splenic hamartomas or typical inflammatory pseudotumors, which lack this characteristic combination of fibrosis and diverse vascular architecture (8, 25–27).

**Figure 3:**
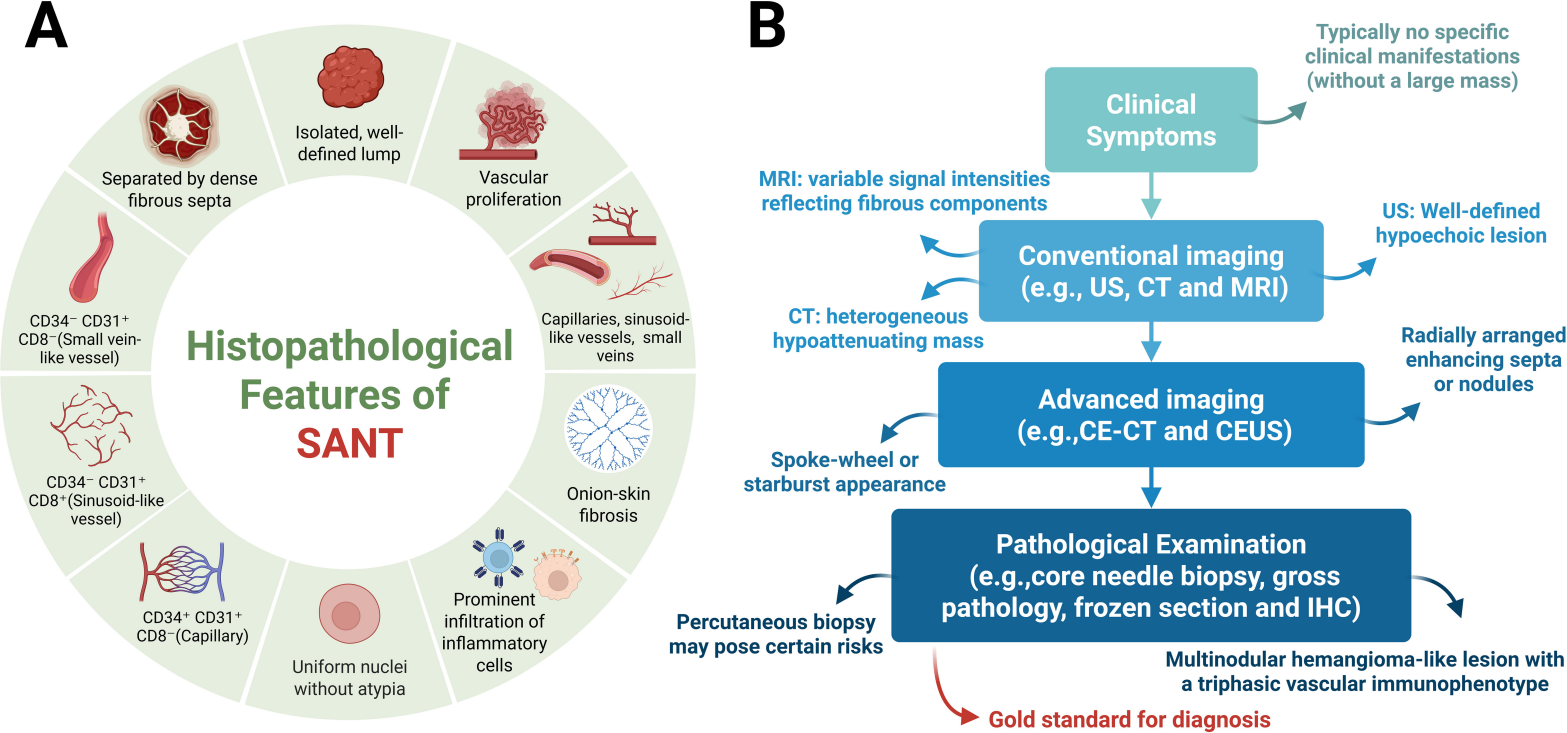
Pathological features and clinical diagnostic workflow of SANT. **(A)** Pathological Features. From a gross pathological perspective, SANT presents as a solitary, well-circumscribed mass within the spleen. On cut surface, the lesion appears as a cluster of coalescent, reddish-brown nodules separated by dense fibrous stroma. Histologically, these nodules consist of proliferative vascular channels, including small slit-like capillaries, larger sinusoid-like vessels, and venule-sized vessels, typically surrounded by concentric rings of collagen (“onion-skin” fibrosis) and varying degrees of inflammatory cell infiltration. At the cytological level, SANT is characterized by highly regular nuclear morphology, with nuclei that are uniform in size and shape and show minimal or absent atypia. Immunohistochemically, SANT exhibits a distinctive tri-phasic vascular profile: (1) capillary-like vessels expressing CD34 and CD31 but not CD8 (CD34^+^/CD31^+^/CD8^-^); (2) sinusoid-like vessels expressing CD8 and CD31 but not CD34 (CD34^-^/CD31^+^/CD8^+^); and (3) venule-like vessels expressing only CD31 (CD34^-^/CD31^+^/CD8^-^). **(B)** Clinical Diagnostic Workflow and Advantages. Clinically, patients with SANT who do not present with massive lesions are often asymptomatic, and the condition is typically discovered incidentally during imaging studies performed for unrelated symptoms or routine health examinations. On imaging, SANT most commonly appears as an incidental splenic mass. In ultrasonography, it generally presents as a well-demarcated, hypoechoic lesion within the splenic parenchyma. Cross-sectional imaging provides further detail: CT scans usually reveal a heterogeneous, hypodense mass, while MRI may demonstrate variable signal intensities reflecting fibrous components. A characteristic radiologic feature of SANT is the “spoke-wheel” or starburst pattern on contrast-enhanced studies, seen as radially arranged enhancing septa or nodules in the arterial phase of enhanced CT or CEUS.Image-guided splenic biopsy is generally avoided due to the organ’s vascularity and fragility, with a high risk of hemorrhage. According to most reports, the diagnostic standard for SANT is surgical excision—either total splenectomy or at least lesionectomy—followed by comprehensive pathological evaluation. Definitive pathological examination remains the gold standard for diagnosing SANT, based on its unique histological characteristics. As previously described, key pathological features include a firm, nodular, sclerotic lesion with a tan-brown cut surface and the presence of multiple angiomatoid nodules with a triphasic immunophenotype. Created with BioRender.com.

Immunohistochemistry (IHC) further contributes valuable diagnostic criteria for SANT, demonstrating a consistent triple-vessel pattern within the nodules, each vessel type characterized by distinct immunophenotypes. Specifically, these include: (1) capillary-like vessels positive for CD34 and CD31 but negative for CD8 (CD34^+^/CD31^+^/CD8^-^); (2) sinusoidal vessels positive for CD8 and CD31 but negative for CD34 (CD34^-^/CD31^+^/CD8^+^); and (3) small vein-like vessels that only express CD31 (CD34^-^/CD31^+^/CD8^-^) (8, 28–32). This tri-lineage vascular expression pattern mirrors the normal composition of splenic red pulp and serves as a hallmark of SANT. Additionally, the endothelial cells within SANT nodules frequently express other vascular markers (e.g., ERG and factor VIII), while the surrounding stromal tissue often highlights smooth muscle actin (SMA) in myofibroblasts (4). Of particular significance is the observation that endothelial cells within SANT lesions generally do not express CD21 or CD35 (markers for follicular dendritic cells) or CD68, a distinction that facilitates the differentiation of SANT from littoral cell angioma of the spleen. Furthermore, in contrast to conventional hemangiomas, which are vascular tumours primarily comprising a single vessel type (typically capillary or cavernous channels with uniformly CD34-positive endothelium) (8). SANT’s distinctive multisclerotic architecture and its characteristic three-vessel immunophenotype (consisting of capillaries, sinusoids, and venules) is pathognomonic. These diagnostic features have been consistently described in multiple case reports and reviews (**Figure 3A**).

### 3.3 Diagnostic approach to SANT

At present, the majority of clinical reports pertaining to SANT characterise diagnoses that are incidental in nature, with the majority of these diagnoses being identified through routine imaging procedures. For patients with clinically suspected SANT, a systematic and sequential diagnostic approach incorporating clinical features (when present), imaging findings, and histopathological analysis is recommended. Initially, patients with non-large SANT lesions generally present without significant clinical discomfort and are typically identified during routine imaging examinations conducted for other health issues or general health screenings. Subsequently, imaging studies usually detect SANT as incidental splenic masses, with each imaging modality discussed individually. For example, on ultrasound, SANT typically manifests as clearly defined hypoechoic lesions within the splenic parenchyma. Cross-sectional imaging provides additional details: CT generally shows heterogeneous low-attenuation masses, whereas MRI may demonstrate variable signals due to fibrous components (8, 33). A notable radiological feature reported for SANT is the “spoke-wheel” enhancement pattern observed in contrast-enhanced studies. During the arterial phase of contrast-enhanced CT or contrast-enhanced ultrasound (CEUS), SANT lesions may exhibit radially arranged enhancing septa or nodules, creating a characteristic spoke-wheel or starburst appearance (34–38), reflecting the vascular nodularity of the lesion. In contradistinction to benign hemangiomas, which characteristically manifest as slow contrast filling and sustained late-phase enhancement, SANT has been observed to enhance more rapidly, thus giving rise to concerns regarding potential malignancy. However, it should be noted that these imaging findings are not entirely specific, and SANT may be misinterpreted as hemangiomas, hamartomas, littoral cell angiomas, or even angiosarcomas (8, 37, 39–41). Indeed, no definitive radiological signs exist to reliably differentiate SANT from other splenic tumors solely based on imaging studies. Due to diagnostic uncertainty and the spleen’s vascular nature, histopathological confirmation is almost always necessary.

Thirdly, tissue sampling is typically performed via needle biopsy (limited tissue), intraoperative frozen section examination (partial), and comprehensive postoperative pathological evaluation. The following sections will discuss these methods in turn. Image-guided splenic biopsies are typically avoided due to the organ’s high vascularity and fragility, which pose significant bleeding risks. Furthermore, biopsy of a vascular malignancy (such as angiosarcoma) could potentially result in tumor dissemination or rupture. Indeed, clinical experts have cautioned about the risk of spontaneous splenic rupture or malignant dissemination associated with uncertain splenic vascular lesions (8, 42–44). Consequently, the risks associated with needle biopsy of SANT likely outweigh the diagnostic benefits, especially considering the inherent risk of misdiagnosis from limited tissue samples. As far as most of the current authors have reported on their experiences with SANT diagnosis and treatment, the standard for obtaining SANT tissue is excisional biopsy, i.e. surgical removal of the spleen (splenectomy) or at least excision of the lesion for thorough pathological examination. Indeed, Most reported cases of SANT have been definitively diagnosed following elective splenectomy or lesion excision for comprehensive pathological analysis. Moreover, laparoscopic splenectomy is often feasible due to the well-defined and exclusively splenic origin of most SANT lesions (45, 46). In cases where the lesion is of a considerable size or there are concerns about potential rupture, the surgical intervention of an open splenectomy may be contemplated. For instance, in this case, the patient exhibited no symptoms, and the splenic mass was of moderate size. Despite imaging suggesting a benign process, we elected to perform a splenectomy to obtain a definitive diagnosis and eliminate the possibility of hidden malignancy or future complications. This approach is consistent with the literature, which emphasises the necessity of histopathological confirmation for SANT, given that a definitive *in vivo* diagnosis is not feasible. Finally, intraoperative frozen section examination appears to be a promising diagnostic tool and has the potential to preserve the spleen. However, this approach is similarly constrained by the amount of excised tissue and the spleen’s propensity for bleeding. Moreover, the expeditious frozen diagnosis of SANT necessitates gross and immunohistochemical analyses within constrained timeframes.

In summary, definitive pathological examination is considered the gold standard for diagnosing SANT due to its distinctive histological features. Key histopathological characteristics include a firm, nodular, fibrotic mass with a chestnut to brownish-tan cut surface and multiple angiomatoid nodules exhibiting a three-phase vascular immunophenotype. Pathologists must also exclude other entities such as littoral cell angioma or hemangioendothelioma/angiosarcoma. Additional supportive features, including uniform nuclear morphology and low mitotic activity (typically a Ki-67 index of <5%), can further assist pathologists in confirming the diagnosis of SANT(**Figure 3B**).

### 3.4 Potential risks and management post-splenectomy

Although splenectomy generally cures SANT, it carries significant potential risks that must be carefully managed. It is widely acknowledged that the spleen performs a pivotal function in immune system regulation and the maintenance of optimal haematological parameters. Its removal or impairment can lead to a range of complications, the most severe of which is an overwhelming post-splenectomy infection (OPSI) (47). Although rare (with an estimated annual incidence in adult splenectomised patients ranging from 0.1% to 0.5%), this rapidly progressive sepsis, usually caused by encapsulated bacteria, has a mortality rate as high as 50-70% (48–52). In addition to infection, asplenic patients are prone to thromboembolic events. Research has demonstrated that patients lacking a spleen are predisposed to an elevated risk of developing venous thromboembolism (VTE), including deep vein thrombosis and pulmonary embolism, as well as arterial thrombosis, which can manifest as coronary artery disease and stroke. The precise mechanisms remain to be fully elucidated, although they may encompass reactive thrombocytosis and altered coagulation factors following splenectomy. It is noteworthy that chronic thromboembolic pulmonary hypertension is recognised as a late complication in some asplenic patients, presumably caused by microthrombi and abnormal pulmonary vascular remodelling over time (53). Splenectomy has also been demonstrated to induce hematological and immunological changes, including the presence of circulating aged or irregular red blood cells (Howell-Jolly bodies) and potential reductions in specific lymphocyte subsets. Collectively, these changes are indicative of “immune dysfunction”. Some evidence even suggests that chronic inflammation or loss of immune surveillance in asplenic individuals may moderately elevate long-term risks for cardiovascular disease and certain malignancies (54). Nevertheless, infection and thrombosis are the most clearly documented risks.

Given these risks, preventive strategies are crucial for every post-splenectomy patient. Current literature and guidelines recommend a multifaceted approach to mitigate complications (47, 54), including:

- Vaccination: All asplenic patients should receive immunization against major encapsulated organisms. This typically includes the pneumococcal conjugate vaccine series (with regular pneumococcal polysaccharide boosters), Haemophilus influenzae type b (Hib) vaccine, and meningococcal conjugate vaccine. Annual influenza vaccination is also recommended, as influenza can predispose patients to secondary bacterial pneumonia. Ideally, these vaccinations should be administered at least two weeks before elective splenectomy, or approximately two weeks postoperatively if the procedure was emergent, to allow immune recovery. Appropriate vaccination has been shown to significantly reduce the risk of OPSI, although it does not entirely eliminate this risk (55, 56).
- Antibiotic Prophylaxis: It is recommended that antibiotic prophylaxis is administered, particularly during the early post-splenectomy period and for paediatric patients. Many centers suggest daily oral penicillin (or an equivalent antibiotic) for at least 1–2 years post-splenectomy in adults and at least until age 5 in children to prevent pneumococcal infections. For patients with additional immunosuppressive conditions or a previous episode of OPSI, lifelong antibiotic prophylaxis may be considered. As an alternative, some clinicians provide a standing prescription for patients to initiate high-dose oral antibiotics immediately at the onset of fever or infection symptoms while awaiting urgent medical evaluation (57).
- Patient Education: It is imperative that patients are educated about their asplenic state and the associated risks. They are instructed to seek prompt medical attention for any febrile illness or severe symptoms, as delayed treatment of OPSI can rapidly become fatal. Many patients are advised to carry medical alert cards or bracelets indicating their asplenic condition. Education encompasses the provision of counsel on the avoidance of animal bites and the implementation of preventive measures when travelling to regions endemic for malaria or other infectious diseases.
- Thrombosis Prevention: It is imperative that perioperative deep vein thrombosis (DVT) prophylaxis is standard practice in conjunction with any abdominal surgery. This prophylaxis should encompass the utilisation of low molecular weight heparin and compression devices, in accordance with established protocols. If there is significant postoperative reactive thrombocytosis (platelet counts > 1,000 × 10^9/L), some experts recommend short-term, low-dose aspirin to mitigate thrombosis risk, although this is individualized (58). In light of the heightened baseline risk, it is imperative for individuals with long-term asplenia to proactively manage modifiable atherosclerotic risk factors. These include maintaining optimal blood pressure and cholesterol levels, and abstaining from smoking. Regular follow-up complete blood counts can monitor persistent thrombocytosis or other hematologic changes. Chronic thromboembolic pulmonary hypertension, a rare complication following splenectomy, requires specialized management by a cardiopulmonary team.

By implementing these preventive measures, patients can benefit from splenectomy (potentially curing splenic lesions like SANT) while minimizing the associated risks of asplenia (54). In our patient’s case, we ensured all appropriate preventive measures were timely administered preoperatively, educated the patient about recognizing infection signs and the necessity of urgent evaluation if infection symptoms appeared, and established perioperative anticoagulation prophylaxis along with scheduled follow-ups to monitor blood counts and overall health. As emphasized by Weledji et al. (54), modern practice stresses spleen-preserving methods where feasible to avoid these risks. However, in the case of SANT—where splenectomy simultaneously provides diagnosis and cure—the consensus is that the procedure is justified provided meticulous post-splenectomy care is in place. Currently, our patient remains well, and with appropriate preventive strategies, the likelihood of severe post-splenectomy complications should remain low.

### 3.5 Therapeutic options amid mechanistic controversy of SANT

At present, the mechanism of SANT is controversial, and the mechanisms is unknown because of its rarity, unknown natural course of development and complexity of pathological phenotypes. The evaluation of the definition and nature of SANT also affects clinicians’ choice of treatment options for SANT patients.

As previously discussed, One view is that SANT is only a reactive, non-neoplastic proliferation secondary to chronic vascular injury or inflammation (1), This perspective proposes that SANT represents an exaggerated reparative response to vascular damage: injury to small splenic vessels or the venous outflow tract may provoke the formation of hemangioma-like vascular nodules, which subsequently undergo fibrous obliteration. The basis of this view is mainly as follows: 1. At present, there is no positive expression of serum conventional tumour markers in patients with SANT, and there is no report of SANT recurrence or metastasis, which may suggest that it is a benign lesion (2). 2. In the pathological section of SANT, apart from the absence of significant mitotic activity and the presence of stable nuclear morphology, the blood vessels showed nodular hyperplasia, that is, micronodular hemangioma-like performance, which may suggest a compensatory phenomenon after injury (6). There were also vimentin- and SMA-positive spindle myofibroblasts in the sections, indicating that the fibrosclerotic interstitial septated hemangioma-like structure shown by this compensatory phenomenon may be caused by the proliferation of myofibroblasts (3). It can be speculated that after the spleen tissue is damaged by inflammation, myofibroblasts proliferate reactively, resulting in the spleen tissue being divided into round-like hemangioma-like nodules. Then, these nodules gradually developed fibrosis in the process of continuous damage healing and eventually appeared as white fibre and scar tissue separated by multiple red nodules in general appearance. In simple terms, SANT may develops from vascular injury or dysfunction, which occurs with the proliferation of blood vessels during healing. 3. Chang et al. (6) evaluated the clonality of X chromosome inactivation in SANT patients (HUMARA) and found that almost all SANT cases were polyclonal. They also found that there is no clinical evidence to prove that SANT is an IgG4-related disease, although some patients are IgG4 positive. Moreover, although some patients were found to contain IPT-like components, their EBER *in situ* hybridization was negative. These findings may mean that SANT is only a polyclonal reactive splenic lesion. It is not a real tumour and has nothing to do with igG4-related diseases and IPT (Inflammatory pseudotumor) (1).

In view of the foregoing, splenectomy may not always be indicated. In the event of a lesion being asymptomatic or associated with mild inflammation, the necessity of splenectomy may be called into question, particularly in light of the spleen’s role in systemic immunity. Splenectomy is not a harmless treatment, and postoperative bleeding, thromboembolism and overwhelming infection after splenectomy have high morbidity and mortality (47, 54).

**Figure 4:**
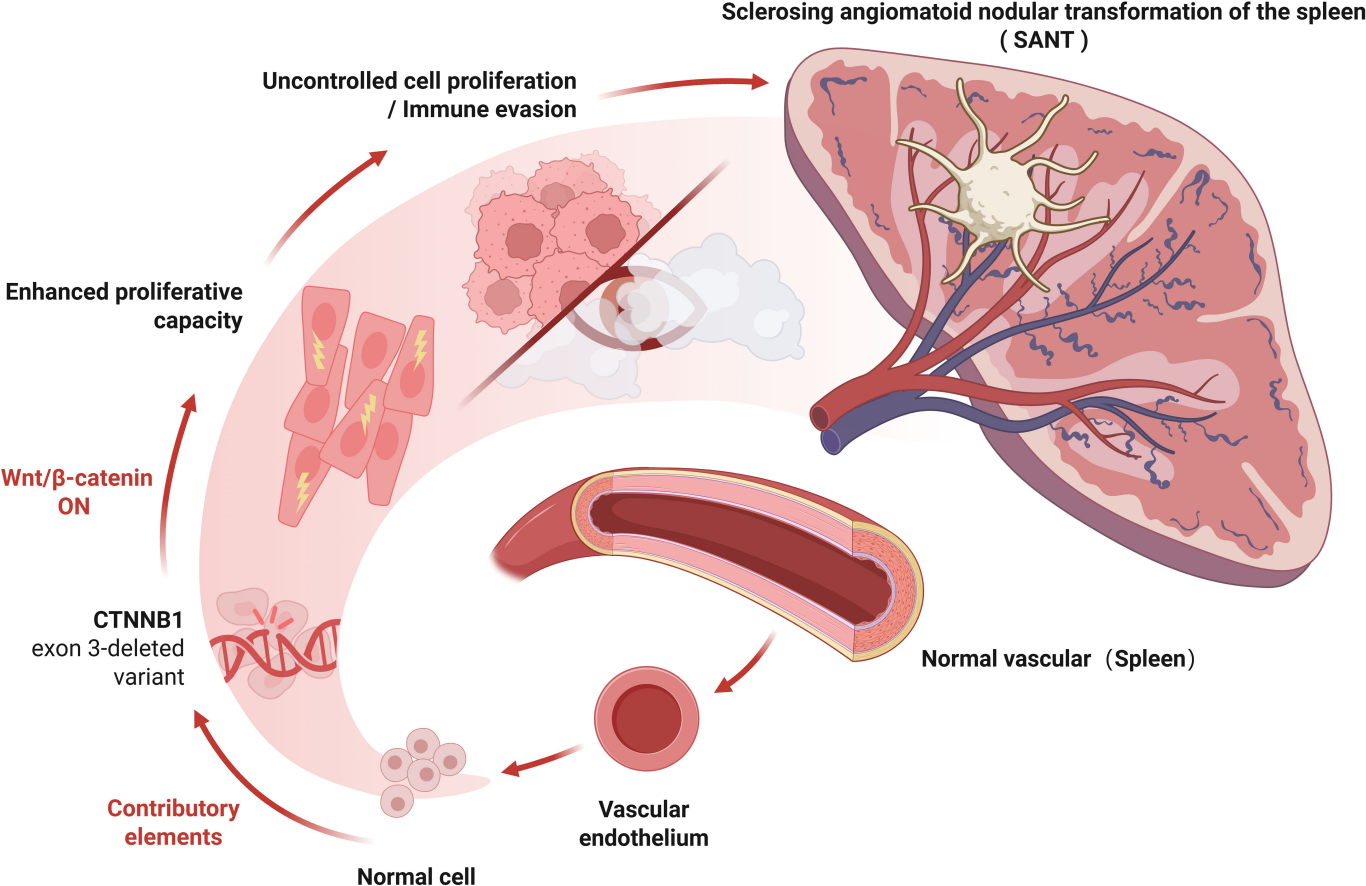
Schematic representation of predicted Wnt/β-catenin pathway activation due to CTNNB1 Exon 3 deletion in SANT. Several cases of SANT have been found to harbor exon 3 deletions in the CTNNB1 gene, which encodes β-catenin. Loss of exon 3 in CTNNB1 prevents β-catenin degradation, resulting in its accumulation and subsequent activation of the Wnt/β-catenin signaling pathway. Aberrant Wnt/β-catenin signaling is a well-established driver in various fibrous vascular tumors and is associated with uncontrolled cellular proliferation. In SANT, stabilized β-catenin may similarly promote the local proliferation of vascular and stromal cells. Moreover, activation of the Wnt/β-catenin pathway can disrupt tumor immune surveillance and facilitate immune escape of neoplastic cells, suggesting a potential for neoplastic transformation within SANT. Created with BioRender.com.

Contrary to the above, another view is that SANT is a precancerous lesion, and surgical removal of the spleen is completely necessary. The main basis is as follows: 1. Many cases have shown that SANT co-occurs with chronic lymphocytic leukaemia, squamous cell carcinoma of lung, colonic carcinoma, early gastric carcinoma and chromophobe renal cell carcinoma, which may suggest a correlation (4). 2. CEUS is a highly sensitive method to distinguish benign and malignant focal lesions (59). In contrast to the persistent late enhancement of benign splenic tumours on CEUS, SANT often shows early arterial spoke-like enhancement (31, 34). This is closer to the manifestation of malignant tumours. 3. A recent fundamental study has provided molecular evidence that lends support to this view. Uzun et al. (2021) reported that multiple cases of SANT harbored exon 3 deletions in the CTNNB1 gene (which encodes β-catenin) (5). It has been established that the deletion of exon 3 in CTNNB1 hinders the degradation of β-catenin, leading to its accumulation and subsequent activation of the Wnt/β-catenin signaling pathway (5).Aberrant Wnt/β-catenin signaling has been identified as a significant driver in various fibrous vascular tumors and is associated with uncontrolled cellular proliferation. In SANT, stabilized β-catenin may similarly promote local proliferation of vascular and stromal cells (60). In addition, activation of the Wnt/β-catenin pathway has immunological consequences: it can disrupt tumor immune surveillance and promote immune escape of neoplastic cells. This prompts the question of whether, within the context of SANT, the lesion may gain a growth advantage through the activation of the Wnt pathway and thereby develop resistance to immune-mediated clearance. Although most current authors regard SANT as a benign entity, the discovery of CTNNB1 mutations may provide indirect evidence suggesting its tumorigenic potential, thereby blurring the distinction between a purely reactive process and a true neoplasm (5). Furthermore, given the present observation of the morphology of SANT, irrespective of its benign or malignant nature, the Wnt/β-catenin pathway-mediated aberrant vascular proliferation, fibrosis, and pro-angiogenic effects may be the principal reasons for the characteristic nodular vascular growth with extensive sclerosis seen in SANT (**Figure 4**).

In this view, limited resection of SANT is considered necessary and beneficial to patients. Moreover, in individual case reports, there were also cases of portal hypertension caused by SANT (43), indicating that SANT may not be as safe as people think, or it may have the possibility of developing other diseases.

In our opinion, whether SANT is a malignant lesion is not the first question to be considered. In contrast, the patient’s current clinical symptoms and whether they can tolerate surgery have a higher priority. If SANT patients are generally in poor condition, the postoperative risk of splenectomy should be avoided to pursue the best curative effect and guarantee the quality of life of patients. In our case, the patient was asymptomatic but could tolerate splenectomy. If SANT is a reactive vasculopathy as the first view suggests, considering the patient’s history of hypertension, diabetes and high triglycerides, these conditions may some of the mechanisms leading to vascular injury. However, even so, we still cannot rule out the possibility that SANT has no risk of malignant transformation or spontaneous rupture of large blood vessels in the natural course of continuous vascular injury and healing. Therefore, we chose splenectomy to ensure that patients had no risk of the above diseases.In addition, it is necessary to supplement the current cutting-edge understanding of the Wnt/β-catenin mechanism and its implications for potential targeted therapies. The main idea is to explore regulation of the Wnt/β-catenin pathway as a strategy to inhibit lesion growth or stromal fibrosis in unresectable cases. In fibrotic disease models, Wnt pathway inhibitors—such as DKK1 or small-molecule Wnt antagonists—have been shown to attenuate myofibroblast activation and pathological collagen deposition, suggesting that Wnt blockade might similarly mitigate the profibrotic component of SANT (61). Likewise, if there is an immune-mediated component in certain cases of SANT—as suggested by reports of IgG4^+^ plasma cell-rich infiltration in some lesions—immunomodulatory therapies (e.g., corticosteroids or other immune modulators) could theoretically influence lesion behavior, although this remains speculative (23). Importantly, despite its clinically benign course and the absence of reported recurrences, the molecular alterations in SANT (i.e., β-catenin pathway activation) position it as a unique fibrous vascular tumor, exhibiting the same signaling features as malignant neoplasms (8). This increases the potential for using SANT as a research model for fibrovascular proliferative diseases, thereby allowing the exploration of antifibrotic and immune-targeted interventions in a controlled benign setting. Viewing SANT through a Wnt-driven pathogenic lens not only deepens our understanding of its etiology but also paves the way for new therapeutic approaches and scientific investigation into the pathogenesis of fibrovascular tumors.

## 4 Conclusion and perspectives

The complex pathological manifestations, unknown natural course of development and rarity of SANT make it difficult for clinicians and pathologists to define its true nature. This report aims to analyze the features and diagnostic workflow of SANT, and to facilitate a more comprehensive understanding by presenting a discussion of two opposing views, thereby highlighting the ongoing contradictions in the literature.

Future research should involve larger-sample, multi-center prospective systematic studies to more fully assess the long-term clinical outcomes of SANT patients, including lesion recurrence rates, risk of malignant transformation, and associated risk factors. In particular, regular long-term imaging follow-up and molecular biomarker testing are recommended, with the goal of earlier identification of possible recurrence or disease progression. Such studies will not only deepen our understanding of SANT pathogenesis, but also provide evidence for establishing more definitive clinical management strategies, thereby better informing clinical decision-making.

## 5 Limitations

Our case report has some limitations. First, the follow-up duration for this case was relatively short (25 months), making it difficult to accurately assess the patient’s long-term prognosis, such as the risk of recurrence or potential for malignant transformation. Second, although current studies generally regard SANT as a benign lesion, the diversity of pathological mechanisms and recent findings of abnormalities in the Wnt/β-catenin pathway suggest there may be as-yet-unidentified long-term risks, including recurrence or malignant potential. Third, the two mainstream opposing views may be influenced by prejudices in different periods and does not characterize the disease. Therefore, a systematic and comprehensive study is needed to help people improve their understanding of the disease to help SANT patients obtain more scientific and effective treatment.

## Data Availability

The original contributions presented in the study are included in the article/supplementary material. Further inquiries can be directed to the corresponding authors.
